# Chiral Lanthanum Metal–Organic Framework with Gated CO_2_ Sorption and Concerted Framework Flexibility

**DOI:** 10.1021/jacs.2c02351

**Published:** 2022-05-03

**Authors:** Francoise M. Amombo Noa, Erik Svensson Grape, Michelle Åhlén, William E. Reinholdsson, Christian R. Göb, François-Xavier Coudert, Ocean Cheung, A. Ken Inge, Lars Öhrström

**Affiliations:** † Chemistry and Biochemistry, Department of Chemistry and Chemical Engineering, Chalmers University of Technology, SE-41296 Gothenburg, Sweden; ‡ Department of Materials and Environmental Chemistry, Stockholm University, Stockholm SE-10691, Sweden; § Nanotechnology and Functional Materials, Department of Material Sciences and Engineering, Uppsala University, SE-751 21 Uppsala, Sweden; ∥ Rigaku Europe SE, Hugenottenallee 167, Neu-Isenburg D-63263, Germany; ⊥ Chimie ParisTech, PSL University, CNRS, Institut de Recherche de Chimie Paris, 75005 Paris, France

## Abstract

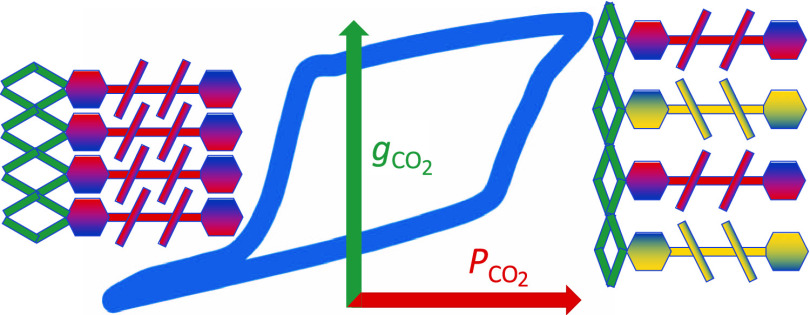

A metal–organic framework (MOF) **CTH-17** based on lanthanum(III) and the conformationally chiral linker 1,2,3,4,5,6-hexakis(4-carboxyphenyl)benzene, cpb^6–^: [La_2_(cpb)]·1.5dmf was prepared by the solvothermal method in dimethylformamide (dmf) and characterized by variable-temperature X-ray powder diffraction (VTPXRD), variable-temperature X-ray single-crystal diffraction (SCXRD), and thermogravimetric analysis (TGA). **CTH-17** is a rod-MOF with new topology **och**. It has high-temperature stability with Sohncke space groups *P*6_1_22/*P*6_5_22 at 90 K and *P*622 at 300 and 500 K, all phases characterized with SCXRD and at 293 K also with three-dimensional (3D) electron diffraction. VTPXRD indicates a third phase appearing after 620 K and stable up to 770 K. Gas sorption isotherms with N_2_ indicate a modest surface area of 231 m^2^ g^–1^ for **CTH-17**, roughly in agreement with the crystal structure. Carbon dioxide sorption reveals a gate-opening effect of **CTH-17** where the structure opens up when the loading of CO_2_ reaches approximately ∼0.45 mmol g^–1^ or 1 molecule per unit cell. Based on the SCXRD data, this is interpreted as flexibility based on the concerted movements of the propeller-like hexatopic cpb linkers, the movement intramolecularly transmitted by the π–π stacking of the cpb linkers and helped by the fluidity of the LaO_6_ coordination sphere. This was corroborated by density functional theory (DFT) calculations yielding the chiral phase (*P*622) as the energy minimum and a completely racemic phase (*P*6/*mmm*), with symmetric cpb linkers representing a saddle point in a racemization process.

## 1 Introduction

Metal–organic framework (MOF) is an excellent term as it is close to a self-definition.^1^ However, it clearly suggests something rigid and static, whereas in reality, MOFs can be “soft”^2^ and display various types of dynamics and flexibility, some very important for their functions.^2−5^ In turn, these may give rise to anomalous bulk material properties such as negative thermal expansion^6^ and negative Poisson’s ratios.^7^

Flexibility is also one of the defining properties that set MOFs apart from other porous materials such as zeolites, mesoporous silica, and mesoporous carbon. The related covalent-organic frameworks (COFs) can also be made flexible,^8,9^ though this seems to be less common. The flexibility is also one reason why gas sorption measurements are more challenging to interpret for MOFs.^10^

While different mechanisms for flexibility in MOFs have been identified,^3,11^ and theoretically investigated,^12^ they mostly rely on the behaviors of individual linkers, metal SBUs, their connection points, and sometimes external triggers (i.e., pressure, temperature, or interaction with guest molecules). Flexibility in MOFs and other porous framework materials such as zeolites can result in an observable “gate-opening” effect, where the accessible porosity of the materials with respect to certain guest molecule increases. It is important, however, not to confuse “gate-opening” with the “breathing effect” such as that observed on MIL-53, even though the two can give similar-looking gas adsorption/desorption isotherms.^13^ Recently, Kitagawa and co-workers reported a MOF where the framework flexibility was modulated through intraframework π–π interactions.^14^ Examples of concerted motion of the entire framework are, however, rare.

**Chart 1:**
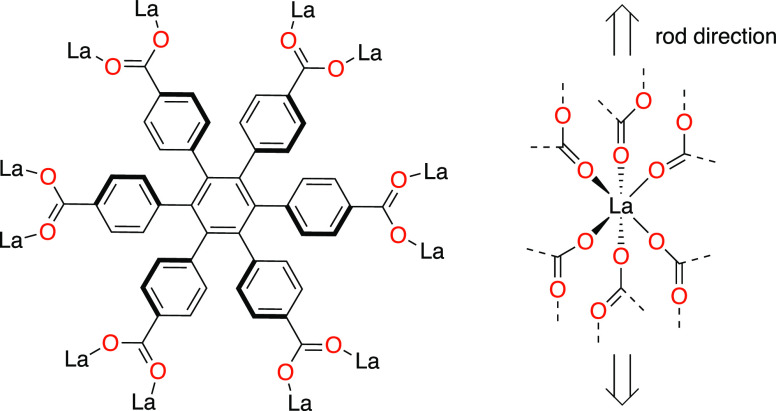
Linker 1,2,3,4,5,6-Hexakis(4-Carboxyphenyl) Benzene, cpb^6–^, Forming **CTH-17**, [La_2_(cpb)]·1.5dmf

It occurred to us that the hexagon-shaped hexatopic linker 1,2,3,4,5,6-hexakis(4-carboxyphenyl)benzene, cpb^6–^ (Chart 1) could display both intra- and interlinker π–π interactions and thus show this new form of flexibility control. Specifically, the conformational propeller-like chirality of cpb could perhaps be transmitted, and even changed, through intraframework π–π interactions and via the coordination sphere.

The cpb linker is one of few flat hexagon-shaped building blocks used in MOF chemistry and probably the only commonly used MOF linker displaying conformational chirality, and potential linker–linker chiral recognition. The one-benzene-ring extended version of cpb, hexakis(4-(4-carboxyl-phenyl)phenyl)benzene, was found in five structures in the Cambridge Crystallographic Database, CSD.^15^ (CEFNIN,^16^ FAKKEK,^17^ FAKKIO,^17^ IXETEM,^18^ and UFOQOY^19^).

For the hexakis(phenyl)benzene core, 10% of structures in the Cambridge Structural Database (CSD) appear to crystallize with one enantiomer only,^20^ corroborating the chiral recognition idea. However, the 14 MOFs prepared to date with cpb have all contained both conformational enantiomers.^20−22^

The relative absence of network topologies based on hexagons has been noted^20^ and motivates this work in terms of reticular chemistry. The earlier observation of frequent two-dimensional (2D) **kgd**-nets with single-metal SBUs and cpb^20^ can be explained by the tris-chelating octahedral geometries at the 3-connected nodes. However, this is not the preferred coordination mode of carboxylates that instead tend to be bridging rather than chelating. Therefore, we theorized that La(III), while providing a neutral [La_2_(pdb)] framework, would not adopt the **kgd**-net with single-metal SBUs.

Our study was triggered by recent developments in three-dimensional electron diffraction (3D ED),^23−25^ enabling structural analysis of nano- and sub-micron-sized crystals, which gave the initial structure solution of the new MOF **CTH-17**.

We were even more intrigued when gas sorption revealed pressure-dependent properties of **CTH-17**. Such behavior indicates flexibility and dynamics, which we further investigated by single-crystal X-ray diffraction at variable temperatures, variable-temperature powder X-ray diffraction, and quantum chemical calculations, suggesting concerted dynamics of the entire framework, an unusual MOF phenomenon.

## 2 Results and Discussion

### 2.1 Synthesis

[La_2_(cpb)]·1.5dmf **CTH-17** was prepared in dimethylformamide (dmf) by the solvothermal method in a steel autoclave. Preparations under milder conditions in a sealed glass tube also afforded various amounts of the formate [La(HCO_2_)_3_] (see the Supporting Information for structure) originating from the breakdown of dmf to formate and dimethylammonium ions, a common occurrence during MOF synthesis.

### 2.2 Structure Analysis of **CTH-17** [La_2_(cpb)]·1.5dmf

Single-crystal data were obtained for **CTH-17**, [La_2_(cpb)]·1.5dmf at 90, 300, and 500 K with Cu Kα radiation λ = 1.540598 Å. Experimental and refinement parameters are given in Table S1 in the Supporting Information. We will discuss the 90 K X-ray structure here, **CTH-17-90K**, and return to the higher temperature structures, when we have presented the results on gas sorption. An overview is found in Table 1.

**Table 1:** Comparison of Selected Experimental and Calculated Structural Parameters for **CTH-17** [La_2_(cpb)]^a^

	SCXRD	SCXRD	SCXRD	PXRD	DFT	DFT
*T* (K)	90	300	500	620		
space gr.	*P*6_1_22	*P*622	*P*622 or *P*6/*mmm*	na	*P*622	*P*6/*mmm*
chiral space gr.^b^	yes	yes	yes or no		yes	no
Flack param.	0.48(3)	0.09(6)	0.49(10)			
*a* (Å)	16.5786(4)	16.5959(16)	16.6393(13)	15.944(2)	16.77	16.46
*c* (Å)	32.213(3)	5.3576(6)	5.2845(8)	10.328(1)	4.96	5.89
*V* (Å^3^)	7667.6(7)	1277.9(3)	1267.1(3)	2274.1(8)	1209	1380
La···La (Å)	5.3753(5)	5.3576(6)	5.2845(8)	5.164^e^	4.96	5.89
void^c^*V* (%)	42	42	42	na	38	45
voids^d^*d* (Å)	4.8	4.8	4.8	na	4.8	4.8; 3.0; 2.8
porosity^f^ (%)	28	29	29		25	38

a Three different crystals were used for X-ray single-crystal diffraction (SCXRD), and we assume the preparations give equal amounts of each enantiomer.

b Chiro-descriptive space group is more precise.

c As calculated by CrystalMaker using van der Waals radii.

d Diameter of largest spheres *r* > 2 Å to fit inside the channels as calculated by CrystalMaker.

e Assuming an isoreticular structure.

f As calculated by Mercury using the kinetic diameter of CO2 and the Contact Surface.

The X-ray single-crystal structure analysis of **CTH-17-90K**, [La_2_(cpb)]·1.5dmf, revealed the chiro-descriptive space group *P*6_1_22 with La^3+^ ions coordinating six nonchelating cpb linkers through one oxygen each, compared to three chelating cpb:s in the **kgd**-nets referred to before. The La-coordination sphere is complemented by disordered dmf molecules giving a total coordination number of La of 7 or 8 (Figure S1). This uneven solvent coordination also causes the large *c* axis. The dmf content is in agreement with the elemental analysis (vide infra).

**Figure 1:**
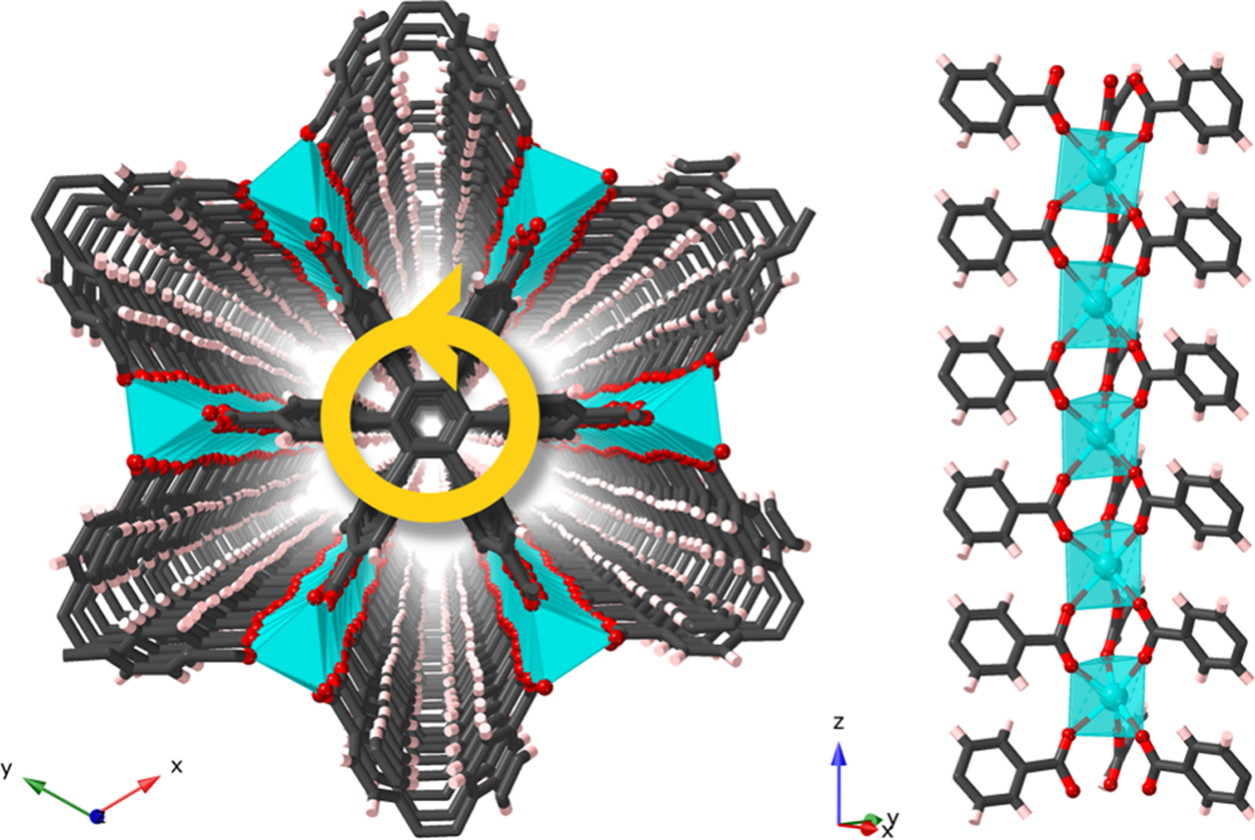
X-ray structure of **CTH-17-90K**, [La_2_(cpb)]·1.5dmf. Left: view along the *z*-axis and the stacked cpb linkers, each connecting to 12 La^3+^ and each linker having the same conformational propeller-like chirality (indicated by the yellow arrow). Right: the LaO_6_ trigonal prisms connected through the carboxylate carbons forming a rod. Disordered dmf molecules have been omitted.

The La coordination results in unbroken rods of LaO_6_ trigonal prisms connected through the carboxylate carbons of cpb and mono- or di-capped by dmf (see Figure 1).

The cpb linkers display the envisaged propeller-like chirality with a same-direction phenyl twist of 60° versus the central benzene ring. For all 17 cpb-like MOFs in the CSD, this twist ranges from 60 to 83° with an average of 71°; see Table S2. In addition, all twists are in the same direction; thus, only one of the chiral conformers is present in the structure model, in agreement with the space group *P*6_1_22.

However, the Flack parameter of 0.48(3) indicates twinning or domains with the opposite chirality *P*6_5_22 for this particular crystal. (The crystal chosen for the 300 K data collection was enantiomerically pure; see below for **CTH-17-300K**, but we expect the bulk material to be made up of equal amounts of crystals with both chiralities.)

**Figure 2:**
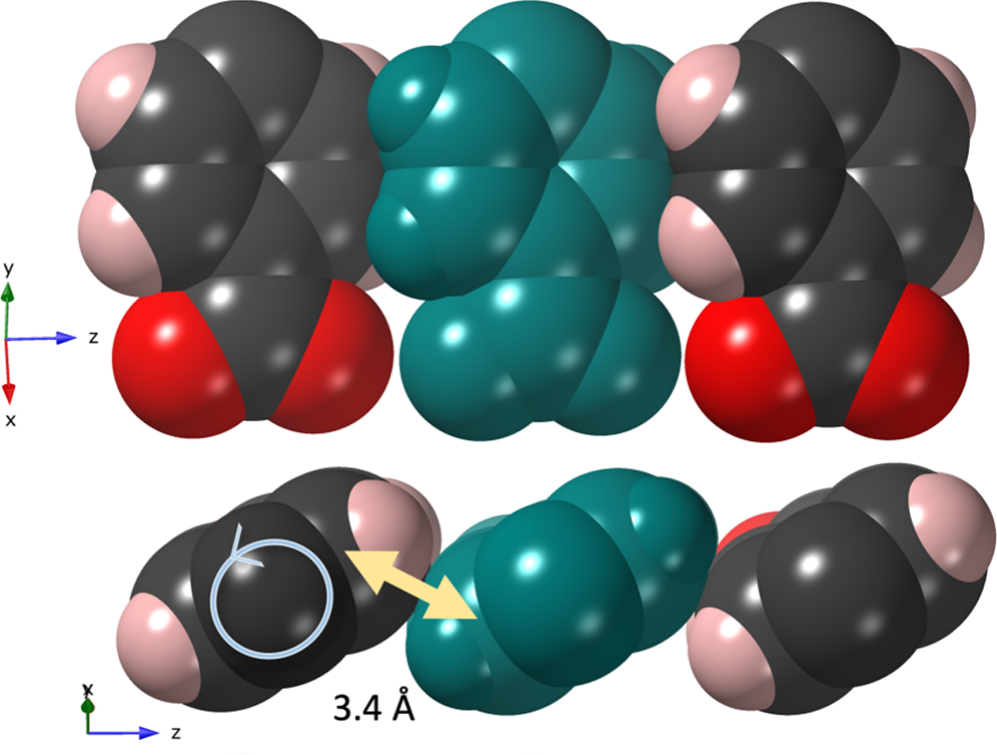
**CTH-17-90K**: two views of the cpb linkers in neighboring molecules using van der Waals radii. They are tightly packed with an average C···C of 3.40 Å as indicated. The bottom view indicates that phenyl group rotation may only be possible by decreasing the phenyl 60° twist.

The cpb linkers are fairly tightly packed (see Figure 2) with closest intermolecular C···C and C···H distances of 3.25(4) and 2.86(3) Å, respectively. This evokes the question if the carboxyphenyl groups are locked in this conformation or if there is an opportunity for movement and if it is then restricted to only one direction. An analysis of the anisotropic displacement parameters (Figure S2) indicates higher values perpendicular to the planes in all benzene rings. The oxygen anisotropic displacement parameters on the other hand are elongated in the direction of the rod (*c* axis). This suggests to us the possibility of a concerted movement of the framework that will be explored in the following sections.

The overall extended structure results in a three-dimensional network with a new rod-MOF topology **och**; see Section 2.9. With all network atoms counted with their van der Waals radii, the void volume calculated from the crystal structure with dmf molecules removed is 38% with oblong channels where spheres having diameters of 4.8 Å can be fitted (the corresponding CO_2_ accessible volume calculated with Mercury^26^ is 28%); see Table 1.

Tables and figures of La coordination bond distance ranges and angles, and the cpb phenyl twist angles for cpb-based MOFs are given in Table S2 and Figure S3.

### 2.3 Gas Sorption Studies

Sorption experiments were carried out after pretreatment of the samples at 275 °C for 6 h in dynamic vacuum (1 × 10^–4^ Pa). Pretreatment at temperatures between 120 and 275 °C was carried out to select the optimal degas conditions. Nitrogen sorption on pretreated **CTH-17** at −196 °C revealed an *S*_BET_ of 231 m^2^ g^–1^. The *S*_BET_ is in the same region as a rough estimate from the crystal structure and modeling tubular channels of diameter 4.8 Å (the diameter of the largest spheres that can be fitted inside the empty MOF): 780 m^2^ g^–1^.

**Figure 3:**
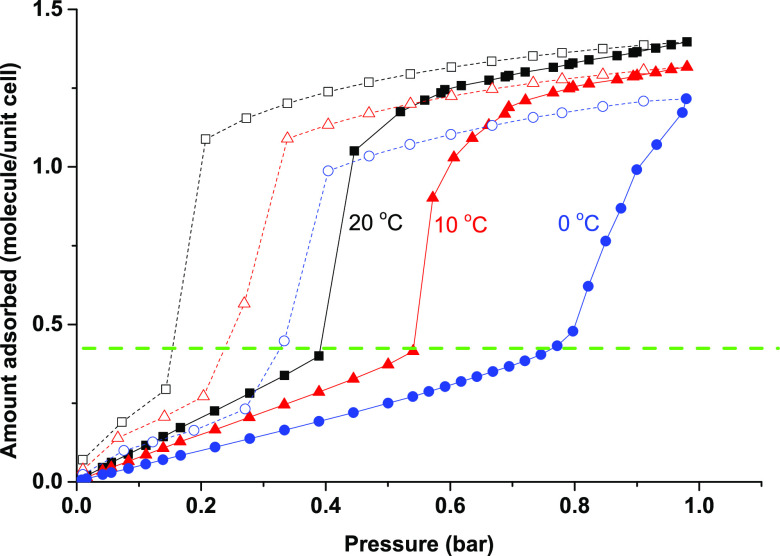
Variable-temperature CO_2_ sorption of activated **CTH-17**. The unit cell refers to the minimal **CTH-17-300K** cell with three channels per unit cell that are accessible to CO_2_.

Narrow pores are sometimes difficult to detect using N_2_ sorption at liquid N_2_ temperature, as sorption kinetics get very slow, and CO_2_ at ambient temperature might be a better alternative.^10^ Moreover, CO_2_ sorption is probably of more practical interest. Therefore, also CO_2_ sorption was tried and immediately revealed a gated-opening behavior (see Figure 3) that prompted us to do an SCXRD variable-temperature experiment.

Figure 3 shows surface sorption up to a certain CO_2_ loading, after which a steep increase in CO_2_ uptake was observed, indicating the opening of some previously inaccessible pores. Desorption hysteresis was observed, and at all temperatures, the gate-opening appeared to be reversible upon desorption of CO_2_. The uptake per minimal unit cell (*c* = 5.36(6) Å, **CTH-17-300K**, see Table 1), excluding what we interpret as surface sorption, is roughly one CO_2_ per unit cell for all temperatures while the calculated space is 3 channels per unit cell (Figure S7).

It appears that this may be due to a gate-opening effect, which is triggered once a CO_2_ pressure is applied when the loading has reached 0.5 CO_2_ per unit cell. Note that gate-opening or breathing effects is not uncommon for MOFs and have been observed by Moon and co-workers on flexMOF,^13^ by Chen and co-workers on SIFSIX-dps-Cu^27^ and is also known of ZIF-7 and ZIF-8.^28−30^ When comparing the shape of the CO_2_ sorption isotherms of **CTH-17** with those shown in these, the apparent shape of the isotherms does resemble those typically observed for MOFs with a known gate-opening effect.

We formulated two hypotheses from this. First that we have an opening up of the structure that depends on the CO_2_ loading (or possibly pressure), and second that this has to do with the dynamics in the structure. We therefore proceeded by determining the X-ray crystal structures also at 300 and 500 K.

### 2.4 Crystal Structures of **CTH-17-300K** and **CTH-17-500K**

**Figure 4:**
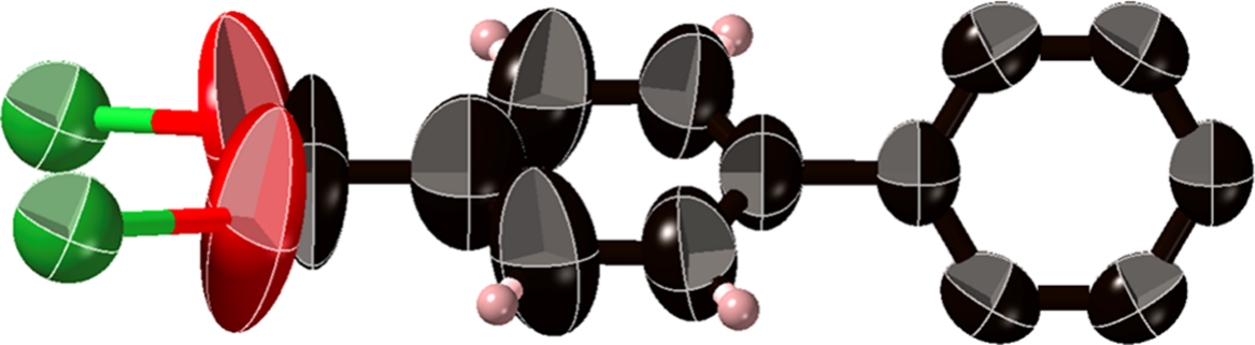
Showing 90% probability ellipsoids indicating phenyl group movement in **CTH-17-300K** at 300 K. The phenyl twists in all structures are 60–61°.

In contrast to the 90 K structure, the X-ray single-crystal structure of **CTH-17-300K** revealed the Sohncke space group *P*622 with a 6-times reduced unit cell axis along the rod-direction, now equal to the La···La distance, and phenyl group movement is indicated in Figure 4. No dmf molecules can be satisfactorily modeled, and the La^3+^ ions thus appear six-coordinated although residual electron density may indicate the presence of dmf. The network is identical to the low-temperature structure, but the *c* axis has been reduced to be the same as the La···La distance. The Flack parameter of 0.09(6) indicates an enantiopure crystal.

The **CTH-17-500K** crystal structure is isostructural to the 300 K, but again the Flack parameter of 0.49(10) indicates twinning or domains with both chiralities. However, at this temperature, the data can equally well be modeled with both enantiomers in a disordered structure and the nonchiral space group *P*6/*mmm*. Therefore, we cannot rule out a solid-state racemization process taking place above 300 K.

A comparison of the structures is given in Table 1, and experimental and refinement parameters are given in Table S1 in the Experimental Section.

### 2.5 Electron Diffraction Structure, **CTH-17**-ED293 at 293 K

Prior to obtaining large enough crystals for X-ray diffraction, a three-dimensional electron diffraction (3D ED) structure determination was performed at 293 K on a 1-μm-sized crystal (Figure S4) led to a similar structure model as later determined by SCXRD in *P*622; see Table S3.

Although 3D electron diffraction can be achieved from nanocrystals about 100 times smaller than required for single-crystal X-ray diffraction, the strong interactions of electrons with matter lead to multiple scattering of electrons in the crystal. These make the intensities of reflections dynamic and cause them to deviate from the kinematic intensities, resulting in high *R*-values from 3D ED data after structure refinement due to the discrepancy between kinematical intensities calculated for the structure model and the dynamical intensities obtained experimentally. This means that Flack parameters cannot be obtained from ED data. The ED data from the particular transmission electron microscopy (TEM) setup that was used also end up giving larger unit cell lengths, and thus larger unit cell volumes, void volumes, porosity, and interatomic distances.

While the observed structural differences per se are not enough to explain the gating behavior, we suggest the motion of the phenyl rings and a potential concurrent stretching of the La rods as an explanation for this pressure- or guest-induced phenomenon.

The rotation of the *tert*-butyl groups in calix[4]arenes has earlier been suggested to trigger the sorption of molecules into seemingly nonporous materials,^31^ and it seems reasonable that such a movement might be set on by gas pressure.

### 2.6 DFT Calculations

The solid-state supramolecular chirality evident from the variable-temperature SCXRD seems to be a very unusual phenomenon,^32,33^ and we, therefore, wanted to firmly establish which structure is the global energy minimum and see what information could be obtained pertaining to the dynamic and high-temperature structures.

The DFT calculations were performed under periodic boundary conditions. The optimization of the larger unit cell starting from **CTH-17-90K** with no solvent molecules relaxes spontaneously to a model with chiral *P*622 symmetry, just as the **CTH-17-300K** structure; see Table 1. The same model is obtained when a *P*6/*mmm* structure is used as the starting point, confirming that the chiral structure is the global minimum.

This also indicates that the larger unit cell and associated space group of **CTH-17-90K** are not intrinsic to the network structure but a result of the coordination of the dmf molecules to La.

Looking in more detail, we see that the DFT-*P*622 model is denser than the experimental structure, a common feature of the DFT methodology in π-stacked structures, due to the overcorrection of the dispersive interactions. The La–O distances are calculated to be 2.43 Å, close to the 2.41(4) Å in **CTH-17-300K** and the 2.39(3)–2.61(8) Å in **CTH-17-90K**. The phenyl twist is 54°, slightly less than the 60° observed experimentally.

While the global energy minimum is relatively easy to model, dynamics is more tricky. We opted instead for a *P*6/*mmm* model, making the phenyl twist exactly 45° and the cpb linker nonchiral. This resulted in an optimized structure with enthalpy 98 kJ/mol (based on [La_2_(cpb)]) higher than that of the chiral *P*622 model.

The *P*6/*mmm* model does not represent the average high-temperature structure, but instead the most symmetric saddle point in the energy landscape when one enantiomer conformation is converted to the other. Neither does it give a good estimate of the activation energy of racemization as the high degree of freedom of the cpb linker means that lower pathways can no doubt be found.

**Figure 5:**
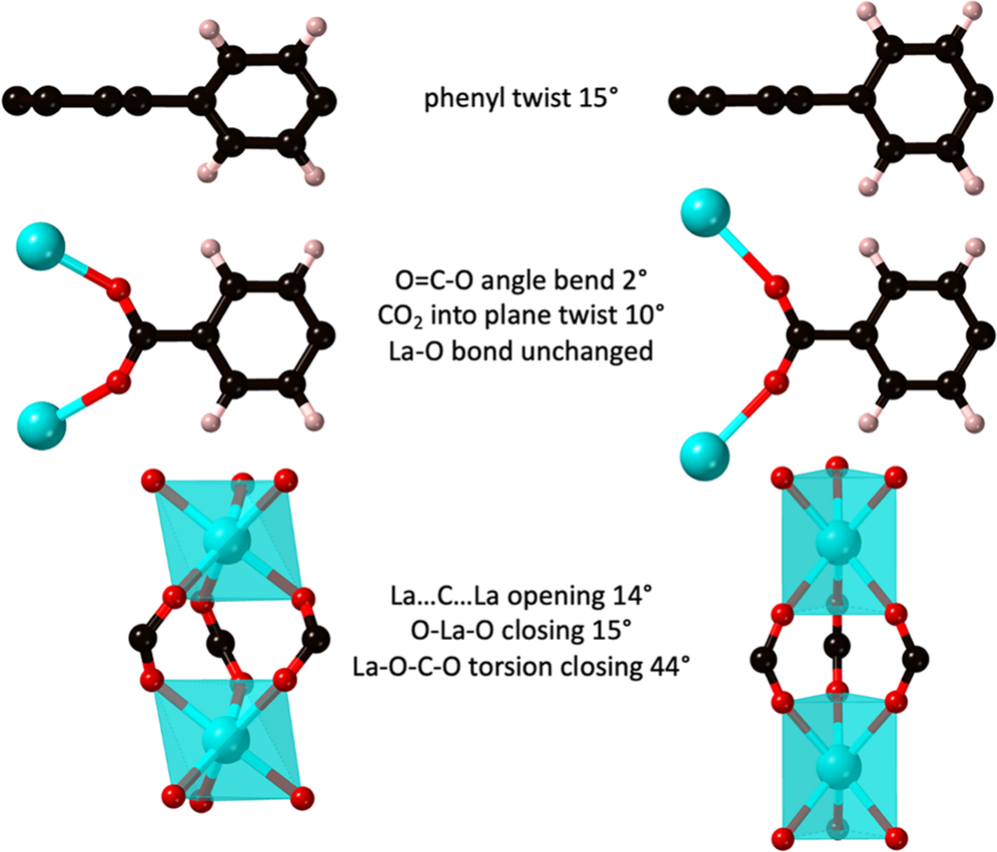
Concerted movements needed to bring about the racemization of **CTH-17**.

It shows that a concerted movement of the entire framework is needed (see Figure 5). The phenyl twist needs more room, so the linkers are separated from each other by an expansion of the rod.

Can this process also generate more space and explain the gas sorption data? Yes it can, because the phenyl twist and the rod elongation that it needs generate more space.

**Figure 6:**
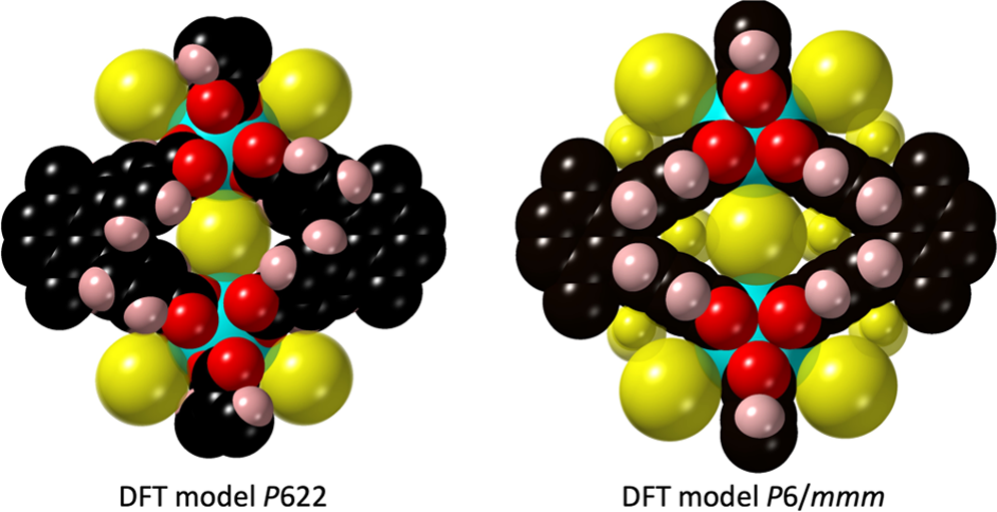
Channels in the DFT models of **CTH-17** (see Table 1) with spheres fitted in the voids. Left: model of the energy minimum. Right: model of a racemization saddle point showing higher void volumes and wider channels.

We can get an idea by comparing the void volumes and channels of the two DFT models. Using the van der Waals radii and accounting for atom overlaps, the total void volume increase is 18% (see Table 1). If we instead look at the cavities accessible to spheres with a radius larger than 1 Å, the effect is higher, almost 40% (see Figure 6).

While these numbers are not absolute, they support the hypothesis that a concerted framework motion can open up the structure sufficiently to give a CO_2_ gating effect.

Another possibility that we need to mention is that CO_2_ coordination could induce structural changes. CO_2_ binding to metal ions has been crystallographically observed before, and in CPO-27, a CO_2_-induced low-temperature structure was observed.^34^

### 2.7 Thermal Stability

Thermal stability assessments of **CTH-17** were made using thermogravimetric analysis (TGA) up to 800 °C and variable-temperature PXRD up to 600 °C. TGA (Figure S5) shows a gradual loss of solvent up to 330 °C and breakdown starting at 380 °C.

**Figure 7:**
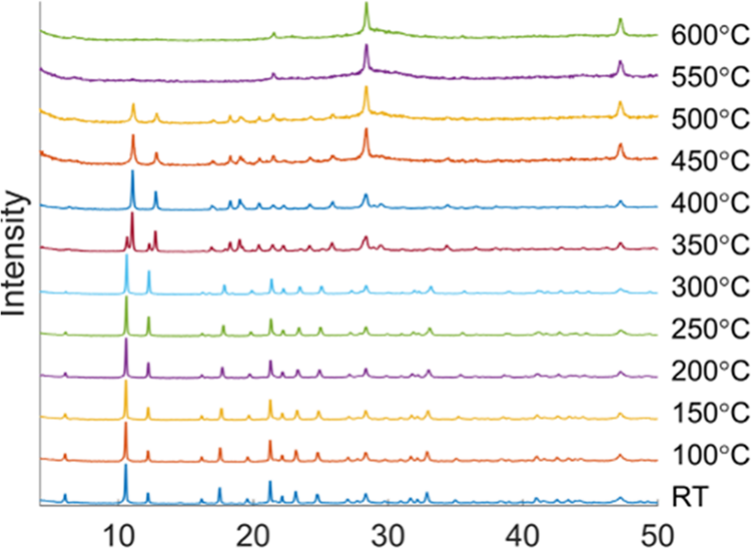
Variable-temperature PXRD of **CTH-17** [La_2_(cpb)]·1.5dmf.

The VTPXRD (Figure 7) shows some gradual peak shifts up to 350 °C, where a structural transformation occurs. The pattern at 400 °C could be indexed to a unit cell with *a* = *b* = 15.944(2) Å, *c* = 10.328(1) Å, α = β = 90°, γ = 120° with the extinction symbol *P*---, which would be consistent with *P*622 and also several other possible space groups (Table S4). This seems to indicate an approximate doubling of the *c* axis compared to **CTH-17-300K** most likely due to a doubled ordering in the structure but with an overall volume contraction of 11% per formula unit. The high-temperature phase **CTH-17-673K** breaks down over 500 °C.

### 2.8 Chemical Stability

The chemical stability of **CTH-17** was probed by soaking the MOF in water at pH 7, aqueous 5 M HCl, and aqueous 5 M NaOH at room temperature for 1 h (Figure S6). **CTH-17** is stable in water for at least 1 h as the corresponding PXRD pattern remains the same as the as-synthesized products albeit with peak broadening. In 5 M HCl, the crystallinity is maintained although some peak broadening was observed, but the final products did not match their as-synthesized diffraction patterns nor any other known pattern, although traces of **CTH-17** may be seen. The lowest chemical stability of **CTH-17** was found when using very basic (5 M NaOH) media. There is a loss of intensity in the PXRD patterns and only traces of crystalline products.

### 2.9 Reticular Chemistry Aspects

**CTH-17** is an example of a rod-MOF^35^ because the metal secondary building unit cannot be reduced to a zero-dimensional (0D) point forming a dot-MOF; instead, it extends in one dimension. However, rod-MOFs pose a problem as they are commonly analyzed in a different way from dot-MOFs.^35−37^ A unified approach was recently proposed.^38^

**Figure 8:**
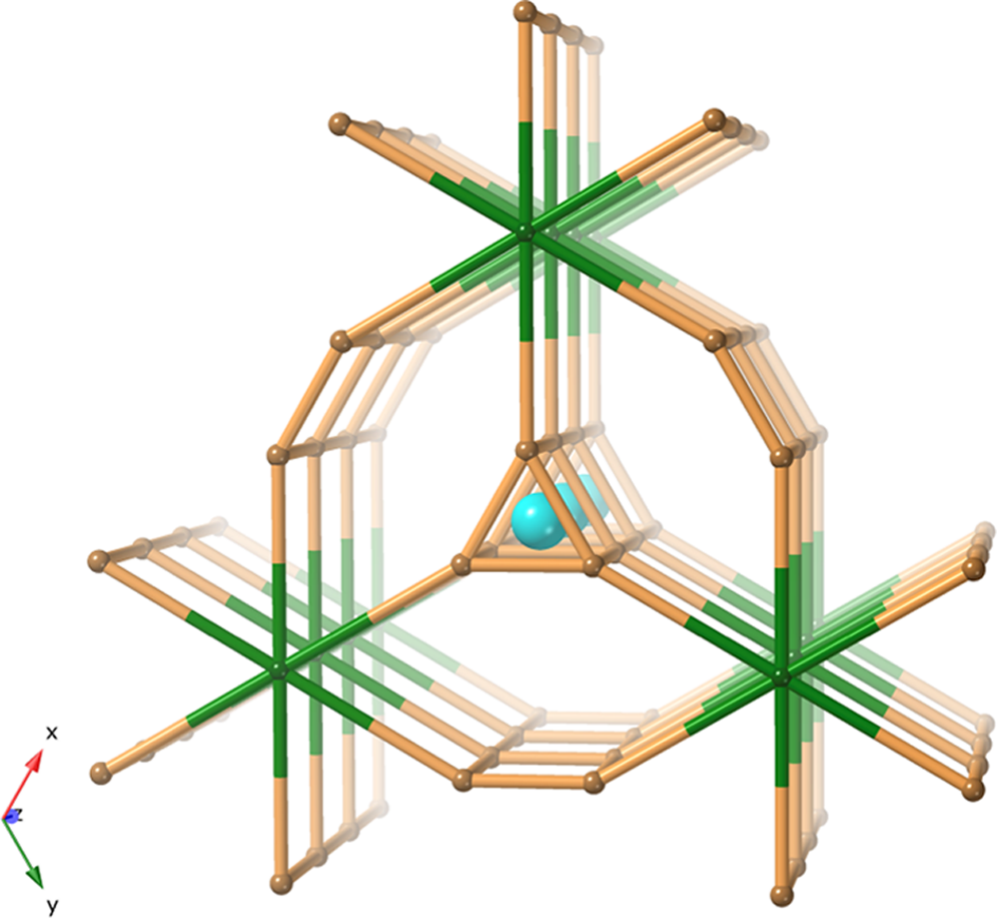
5- and 6-Connected **och-**net by the points-of-extension (PE) method has point symbol {3.4^4^.6^5^}_6_{6^15^}. Lanthanum ions were added for the emphasis of the rod.

For **CTH-17**, this “points-of-extension” (PE) approach gives a 5- and 6-connected net with face-sharing trigonal prisms (see Figure 8). This new **och**-net has the point symbol {3.4^4^.6^5^}_6_{6^15^}. Details of the topology assignment can be found in the Supporting Information.

## 3 Conclusions

We have demonstrated that the rod-MOF **CTH-17**, [La_2_(cpb)]·1.5dmf, is a chiral MOF, the chirality emerging from the stacking of chiral propeller-like cpb linkers. **CTH-17** can appear as enantiopure crystals, and DFT calculations confirm the chiral structure as the energy minimum. However, crystals may also appear twinned, or with domains of opposite chirality, and most likely crystals of both enantiomers are formed in equal amounts in each batch.

**CTH-17** displays a marked pressure-triggered gating effect for CO_2_ loading with a hysteresis of 0.5 bar. We attribute the gating effect to a concerted movement of the entire framework that can potentially increase the available space for CO_2_ with up to 40% as demonstrated by a DFT model.

Such concerted movement of rods, connectors, and linkers is a very rare occurrence. We note that this may also lead to racemization through scrambling or inversion of the cpb linker conformations. The DFT calculations indicate the saddle point for inversion to be quite high, but we cannot rule out that racemization is happening for the empty MOF at 500 K as diffraction data at this point are ambiguous.

In general, gate opening has been seen triggered by CO_2_ sorption,^13^ and though we have not investigated the selectivity of **CTH-17**, such behavior is useful for applications in renewable energy. Further studies with the one-benzene-group extended hexakis(4-(4-carboxyl-phenyl)phenyl)benzene linker will reveal if we can produce similar structures but with higher CO_2_ capacity.

If gate opening is associated with the change of unit cell parameters, we note that this enables coupling of mechanical and macroscopic properties to chemical properties.

## 4 Experimental Section

### 4.1 Materials and General Procedures

All chemicals utilized for MOF synthesis were purchased from Sigma-Aldrich and were used without further purification. All MOF preparations have been performed repeatedly, and yields are, in general, high, close to quantitative with respect to the metal ion. No independent analysis was performed to confirm the presence of protonated dimethyl amine as the solvent dimethylformamide (dmf) is easily hydrolyzed to dimethyl amine and formic acid. These impurities are always present in dmf unless it is freshly purified and dry. The hydrolysis is particularly efficient under basic conditions but can also occur under acidic conditions, especially if catalyzed by metal ions or other Lewis acids.^39^ All studied single-crystal MOFs were washed and immersed in dmf before conducting single X-ray diffraction analysis (SCXRD) to remove any remaining unreacted H_6_cpb.

#### 4.1.1 1′,2′,3′,4′,5′,6′-Hexakis(4-carboxyphenyl)benzene (H_6_cpb)

The synthesis of the H_6_cpb was performed as previously reported by Nguyen et al.^21^

#### 4.1.2 [La_2_(cpb)]·1.5dmf **CTH-17**

This compound can be prepared both in a capped pyrex tube for solvothermal synthesis at 120 °C and in a Teflon-lined steel autoclave. The latter procedure tends to give less [La(HCO_2_)_3_] byproduct. H_6_cpb (0.1 g, 0.125 mmol) was dissolved in 40 mL of dmf under stirring at 120 °C in a glass beaker. Once heated, 0.2165 g of 0.5 mmol lanthanum nitrate hexahydrate (La(NO_3_)_3_) and 10 mL of acetic acid were added, and the mixture was stirred. When a clear solution was obtained, the solution was transferred to a Teflon-lined, stainless-steel autoclave, which was then transferred to a preheated Memmert UN75plus oven at 150 °C. The oven was programmed to maintain heat for 10 days, and then the temperature was gradually decreased to room temperature. After the heating program was finished, a white precipitate had formed, which was filtered and then washed three times with 10 mL of dmf, and after drying at room temperature, 118.4 mg (yield of 80.0%) of a white powder with small white crystal was obtained suitable for SCXRD. Elemental analysis C_52.5_H_34.5_La_2_N_1.5_O_13.5_ calculated (found): C 53.43 (53.13); H, 2.95 (2.69); N, 1.78 (1.19).

#### 4.1.3 Electron Diffraction

3D electron diffraction data of **CTH-17** were collected using a JEOL JEM-2100 TEM, equipped with a Timepix detector from Amsterdam Scientific Instruments, while continuously rotating the crystal at 0.45° s^–1^.^40,41^ Data reduction was performed using XDS,^42^ and the structures were subsequently solved using ShelXT.^43^

#### 4.1.4 Single-Crystal X-ray Diffraction

Data were collected using Cu Kα radiation (λ = 1.54184 Å) on a Rigaku XtaLAB Synergy-DW diffractometer equipped with a HyPix-Arc 150° detector and on a Synergy-R, diffractometer equipped with a HyPix-Arc 150° detector. Diffraction data were acquired and processed with CrysAlisPro software package.^44,45^ Direct or structure expansion methods were used for all structures, and the refinements were established by full-matrix least squares with SHELX-2018/3^46^ using X-seed^47^ and Olex2^48^ software as a graphical interface. Details of structure refinements are found in the Supporting Information.

#### 4.1.5 Powder X-ray Diffraction

Powder X-ray diffraction patterns were recorded using a Bruker D8 Twin diffractometer (Billerica, Massachusetts) with Cu Kα radiation λ = 1.54 Å at room temperature scanning between 2θ = 0 and 50°. Variable-temperature powder X-ray diffraction data were collected using a Panalytical X’Pert Pro diffractometer (Cu Kα_1,2_, λ_1_ = 1.540598 Å, λ_2_ = 1.544426 Å) using a Bragg–Brentano geometry, equipped with an Anton Paar XRK 900 high-temperature chamber.

#### 4.1.6 Other Tools

Elemental analysis was performed by Mikroanalytisches Labor Kolbe, c/o Fraunhofer Institut, Oberhausen, Germany. For TGA measurements, we used a Mettler Toledo TGDS/DSC 3+ Star system. CrystalMaker was used for all structure drawings and porosity and cavity calculations. For the two latter calculations, van der Waals radii were used. CrystalMaker calculates empty volumes as the residual volume after atomic volumes have been subtracted from the total volume. For the size of cavities, CrystalMaker scans through the structure, aiming to find the largest sphere that can fit into any cavity using multiple iterations.^49^ Systre was used for topology analysis.^50^ We also used Mercury to calculate voids using Contact Surface giving the volume that can be occupied by a probe of a given radius, in this case 1.6 Å corresponding to the kinetic diameter of CO_2_. The Cambridge Crystallographic Database 5.42 was used for the data in Table S2 and Figure S3.^15^

N_2_ and CO_2_ adsorption isotherms were recorded on a Micromeritics ASAP2020 surface area analyzer at liquid N_2_ temperature (−196 °C). The samples were pretreated up to 275 °C under dynamic vacuum (1 × 10^–4^ Pa) for 6 h before the analysis. The relative pressure range of 0.05–0.15 was used to estimate the Langmuir and Brunauer–Emmett–Teller (BET) surface area of the samples. Additionally, CO_2_ and N_2_ adsorption isotherms were recorded at 0, 10, and 20 °C (with a temperature- controlled water bath) using the same instrument.

Density functional theory (DFT) calculations were performed with the CRYSTAL17 code.^51^ The basis sets used were taken from the online CRYSTAL basis sets library (https://www.crystal.unito.it/basis-sets.php): all-electron Gaussian basis sets of double-ζ valence with polarization for O, C, and H;^52^ an effective core potential was used for La.^53^ The exchange-correlation functional chosen was the PBE functional^54^ and a Grimme “D3” dispersion correction.^55^ Representative input files for the calculations are available online at https://github.com/fxcoudert/citable-data

## Supporting Information Available

General procedure, SCXRD crystallographic information of the MOFs, crystallographic information for the 3D ED data, thermal analysis, PXRD Chemical stability studies, and network topology (PDF)

## Accession Codes

CCDC 2155881–2155884 and 2155886 contain the supplementary crystallographic data for this paper. These data can be obtained free of charge via www.ccdc.cam.ac.uk/data_request/cif, or by emailing data_request@ccdc.cam.ac.uk, or by contacting The Cambridge Crystallographic Data Centre, 12 Union Road, Cambridge CB2 1EZ, UK; fax: +44 1223 336033.
