# Supplementary material for: Chiral Lanthanum Metal–Organic Framework with Gated CO2 Sorption and Concerted Framework Flexibility
Source: J Am Chem Soc. 2022 May 3;144(19):8725–33. doi: 10.1021/jacs.2c02351 (PMC9122260; doi:10.1021/jacs.2c02351)
Supplement: Supplementary file 1 — ja2c02351_si_001.pdf [file ja2c02351_si_001.pdf]

Supporting Information

A chiral lanthanum Metal-organic Framework with gated CO_2 Sorption and concerted framework flexibility

Francoise M. Amombo Noa,[a*] Erik Svensson Grape,[b] Michelle Åhlén,[c] William E. Reinholdsson,[a] Christian R. Göb,[d] François-Xavier Coudert,[e] Ocean Cheung,[c] A. Ken Inge,[b] Lars Öhrström[a*]

[a] Chemistry and Biochemistry, Dept. of Chemistry and Chemical Engineering, Chalmers University of Technology, SE-41296 Gothenburg, Sweden; [b] Department of Materials and Environmental Chemistry, Stockholm University, Stockholm SE-10691, Sweden; [c] Nanotechnology and Functional Materials, Department of Material Sciences and Engineering, Uppsala University, SE-751 21 Uppsala, Sweden; [d] Rigaku Europe SE, Hugenottenallee 167, D-63263 Neu-Isenburg, Germany. [e] Chimie ParisTech, PSL University, CNRS, Institut de Recherche de Chimie Paris, 75005 Paris, France

General procedure 2
SCXRD crystallographic information of the MOFs 3
CSD analysis of cpb-MOFs and La-carboxylates 5
Transmission electron microscopy 6
Crystallographic information for the 3D ED data 6
Thermal analysis 7
PXRD Chemical stability studies 8
Network topology 9

General procedure

Thermogravimetric analysis (TGA). TGA were carried out on a Mettler Toledo TGA/DSC3+ in air atmosphere with a heating rate of 10 ^{0}C/min. The samples were analyzed between 30 ^{0}C and 800 ^{0}C.

Synthesis of MOFs

Two routes were utilized for the synthesis of CTH-17. One route was performed in a glass vial and left in an oven at 150 ^{0}C until crystals were formed. The second method utilized a Teflon-lined stainless-steel autoclave and was put in OVEN (Memmert UN75plus) and a specific heating and cooling programme was used.

For the first method, 10 mg (0.0125 mmol) of H_6cpb was dissolved in 5 ml of DMF under stirring at 120°C in a glass vial. Once heated, 21.65 mg (0.05 mmol) of lanthanum nitrate hexahydrate ($La(NO_3)_3$), and 1 ml of glacial acetic acid was added, and the mixture was left to stir. When a clear solution was obtained, the vial was capped transferred to an oven and heated at 150°C for 7 days, at which point a white precipitate was formed. The white precipitate was filtered, and then washed 3 times with 5 ml of DMF. After drying in room temperature, a white powder with small white crystal was obtained.

For the second aforementioned method, 0.1 g (0.125 mmol) of H_6cpb was dissolved in 40 ml of DMF under stirring at 120°C in a glass beaker. Once heated, 0.2165 g (0.5 mmol) of lanthanum nitrate hexahydrate ($La(NO_3)_3$), and 10 ml of glacial acetic acid was added, and the mixture was left to stir. When a clear solution was obtained, the solution was transferred to a Teflon-lined, stainless-steel autoclave, which was the transferred to a preheated Memmert UN75plus oven at 150°C. The oven was programmed to maintain heat for 10 days, the gradually decrease the temperature back down to room temperature. After the heating programme was finished, a white precipitate had formed. The white precipitate was filtered, and then washed 3 times with 10 ml of DMF, and after drying in room temperature, a white powder with small white crystal was obtained. The powder appeared more crystalline compared to the product from the vial synthesis, and it was confirmed that less La-formate had formed during the autoclave synthesis. Yields are around 80%.

SCXRD crystallographic information of the MOFs

Table S1. Crystallographic data and structure refinement parameters for CTH-17-90K, CTH-17-300K and CTH-17-500K and $[La(HCO_2)_3]$.

Code	CTH-17-90K	CTH-17-300K	CTH-17-500K	$[La(HCO_2)_3]$
Structural formula	$C_{57}H_{24}N_3O_{15}La_2$	$C_{24}H_{12}O_6La_1$	$C_{24}H_{12}O_6La_1$	$C_3H_3O_6La_1$
Molecular mass (g mol^{-1})	1268.61	535.25	535.25	273.96
Data collection temp. (K)	90 (2)	299.98 (10)	500 (2)	100.01 (10)
Crystal system	Hexagonal	Hexagonal	Hexagonal	Trigonal
Space group	$P6_122$	*P*622	*P*622	*R*3*m*
a (Å)	16.5786(4)	16.5959(16)	16.6393(13)	10.7247 (3)
b (Å)	16.5786(4)	16.5959(16)	16.6393(13)	10.7247 (3)
c (Å)	32.213(3)	5.3576(6)	5.2845(8)	4.1382 (2)
α (°)	90	90	90	90
β (°)	90	90	90	90
γ (°)	120	120	120	120
Volume (Å^3)	7667.6(7)	1277.9(3)	1267.1(3)	412.2(3)
Z	6	2	2	3
Dc, calc density (g cm^{-3})	1.648	1.391	1.403	3.311
Absorption coefficient (mm^{-1})	13.367	13.192	13.272	59.967
θ range	3.37-64.97	3.01-53.63	6.14-51.25	8.16-68.83
Reflections collected	23224	3657	746	1143
No data I >2 sigma (*I*)	2158	556	367	195
Final *R* indices [I >2 sigma (*I*)]	R_1 = 0.0817 wR_2 = 0.1966	R_1 = 0.0759 wR_2 = 0.2289	R_1 = 0.0955 wR_2 = 0.2479	R_1 = 0.0180 wR_2 = 0.0441
R indices (all data)	R_1 = 0.1549 wR_2 = 0.2399	R_1 = 0.1036 wR_2 = 0.2502	R_1 = 0.1001 wR_2 = 0.2533	R_1 = 0.0180 wR_2 = 0.0441
Goodness-of-fit on F^2	1.001	1.143	1.126	1.134
CCDC no.	2155881	2155883	2155884	2155886

* Kistaiah et al earlier reported the cell parameters from an X-ray powder diffraction study,[1] and Bolotovsky et al. also reported the coordinates from a neutron powder diffraction study[2]. Our data are in agreement with these studies but also provide single crystal X-ray diffraction quality.

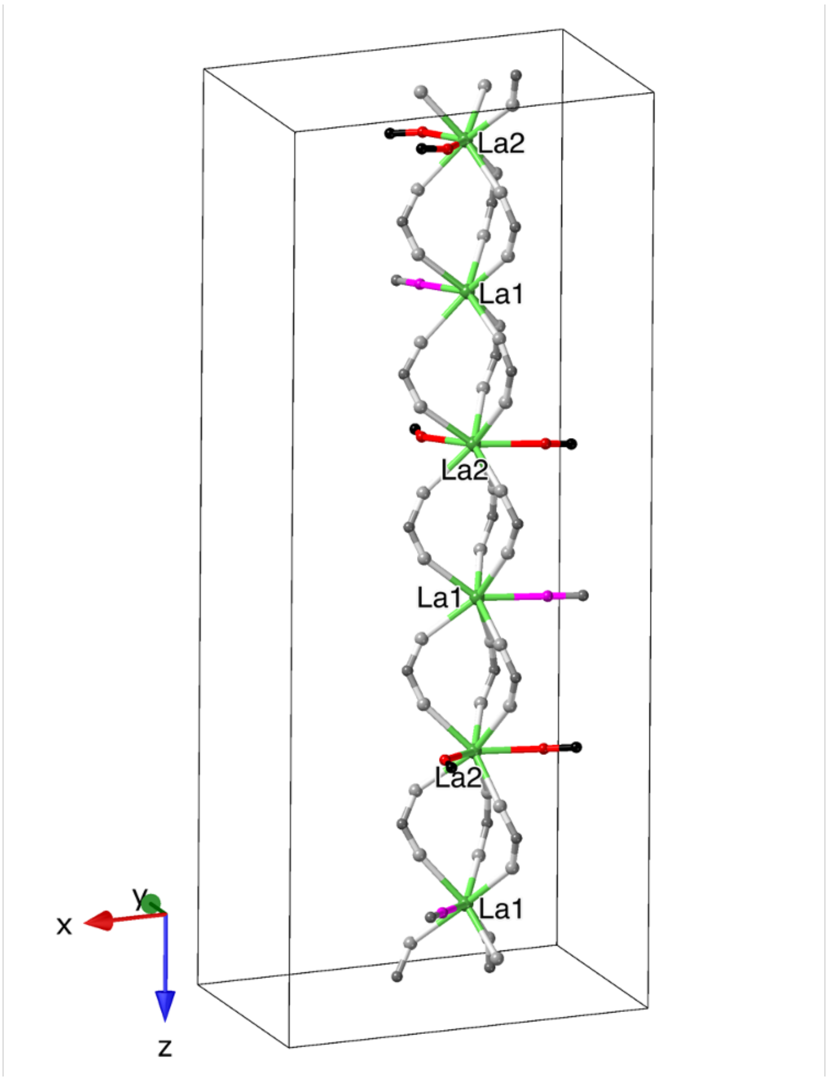

Figure S1. The helical distribution of the coordinated dmf molecules (red/black or pink/black for the two independent dmf:s) in **CTH-17-90K** responsible for the long c-axis.

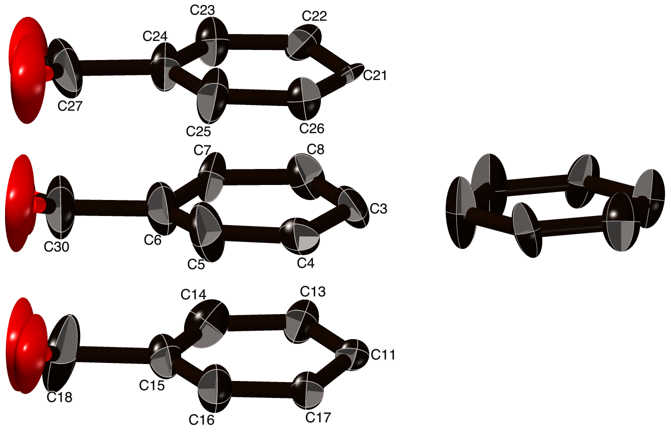

Figure S2. 50% Ellipsoid plot of the crystallographic independent phenyl groups in the 90K structure (left) and the central benzene ring (right)

CSD analysis of cpb-MOFs and La-carboxylates

Table S2. Phenyl twist angles for cpb-MOFs and related compounds, grand average is 70.8°

Refcode	average twist angle(°)	standard deviation of twist angles (°)
AZAVII	67.3	9.7
AZAVOO	68.9	4.8
AZAVUU	75.7	5.7
AZAWAB	76.4	5.8
AZAWAB	72.9	9.8
CUSQEP	82.7	2.4
CUSQIT	64.6	6.5
CUSQOZ	77.8	2.7
CUSQOZ	76.8	7.1
CUSQUF	75.5	7.9
CUSQUF	79.0	2.6
CUSRAM	63.8	5.0
CUSRAM	64.0	4.8
CUSREQ	69.0	4.4
CUSTIW	60.4	1.7
NIJSOQ	64.3	4.4
NIJSOQ	65.9	3.8
NIKGIZ	67.1	9.1
NIKGOF	68.1	5.8
NIKGOF	69.3	6.3
NIKGUL	65.5	4.2
NIKGUL	66.9	4.8
OFOJIF	64.7	2.2
XAGLAW	75.8	8.9
XAGLAW01	75.8	8.8

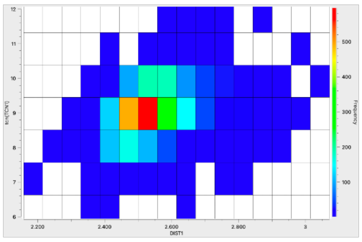

Coordination number versus La-O distance for lanthanum carboxylates. Clearly the La-O bond length increases with coordination number

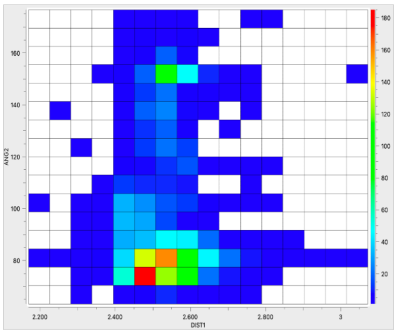

La-C-La angle versus La-O distance for La-O-C-O-La carboxylates. Shorter La-O seems to correlate with smaller La-C-La angle. Opening up the structure needs a larger La-C-La angle as indicated by our DFT calculations.

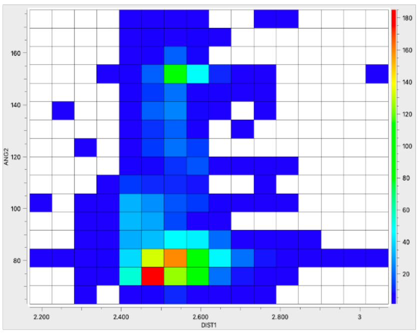

La-C-La angle versus La-O distance for La-O-C-O-La carboxylates. Shorter La-O seems to correlate with smaller La-C-La angle. Opening up the structure needs a larger La-C-La angle as indicated by our DFT calculations.

Figure S3. CSD data for La-carboxylates.

Transmission electron microscopy

Scanning electron microscopy image of CTH-17-ED293K was collected on a JEOL JEM-2100 TEM.

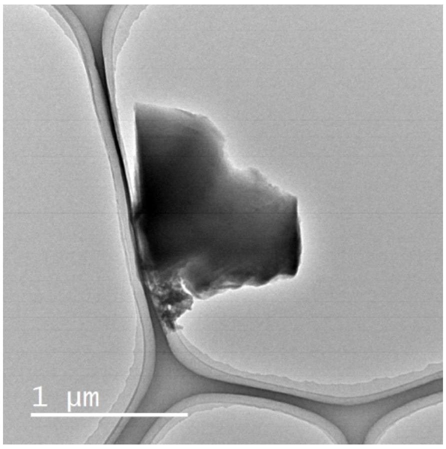

Figure S4. TEM micrographs of CTH-17-ED293K.

Crystallographic information for the 3D ED data

Table S3. Crystallographic information for the 3D ED data of CTH-17-ED293K crystals.

Identification code	CTH-17-ED293K, crystal 1
Crystal system	Hexagonal
Space group	*P*622 (No. 177)
Unit cell dimensions	a = 17.220(2) Å
	b = 17.220(2) Å
	c = 5.5100(11) Å
	$\alpha = 90^0$
	$\beta = 90^0$
	$\gamma = 120^0$
Volume (Å^3)	1415.0(5) Å^3
Z	2
Rotation range	77.84° (-52.84 to 25.00°)
Index ranges	$-20 \le h \le 21$
	$-21 \le k \le 21$
	$-6 \le l \le 6$
Reflections collected	20414
Independent reflections	985
	[R(int) = 0.1977]
Completeness (to 1.1 Å resolution)	100 %
R_1 (ED model) [$I > 2\sigma(I)$]	0.1349
CCDC no.	2155882

Table S4. Crystallographic table for the Pawley refinement of **CTH-17-673K** against PXRD data acquired at 400 °C in air.

Compound	CTH-17
Crystal system	Hexagonal
Space group	*P*622 (No. 177)
Unit cell dimensions	a = 15.944(2) Å
	c = 10.328(1) Å
Volume (Å^3)	2274.1(8) Å^3
Wavelength	λ_1 = 1.540598 Å
	λ_2 = 1.544426 Å
Refinement method	Pawley
Refinement statistics	R_{wp} = 7.68 %
	GOF = 0.92

Thermal analysis

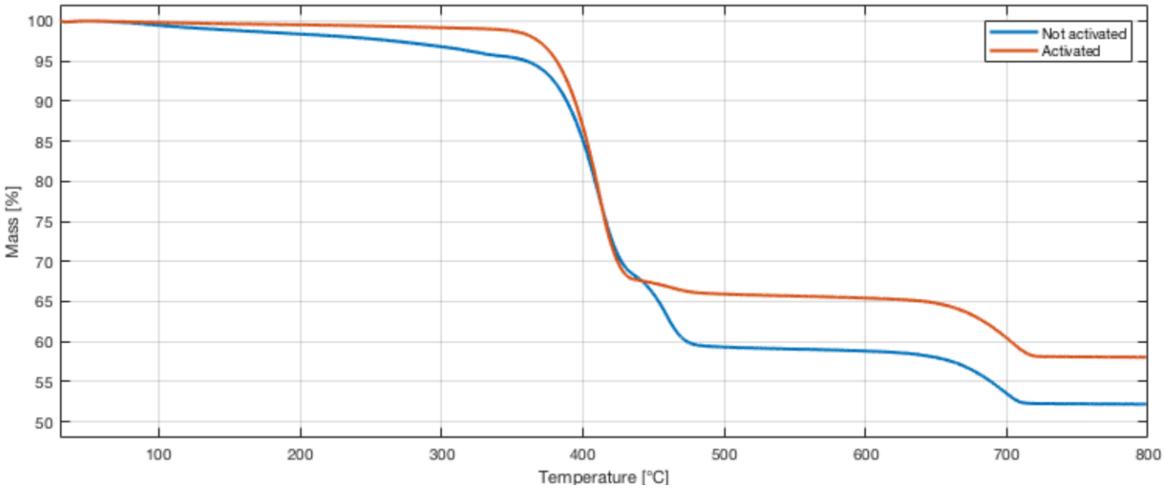

Figure S5. TGA traces of the as synthesized (blue) and activated (orange) LaMOF.

PXRD Chemical stability studies

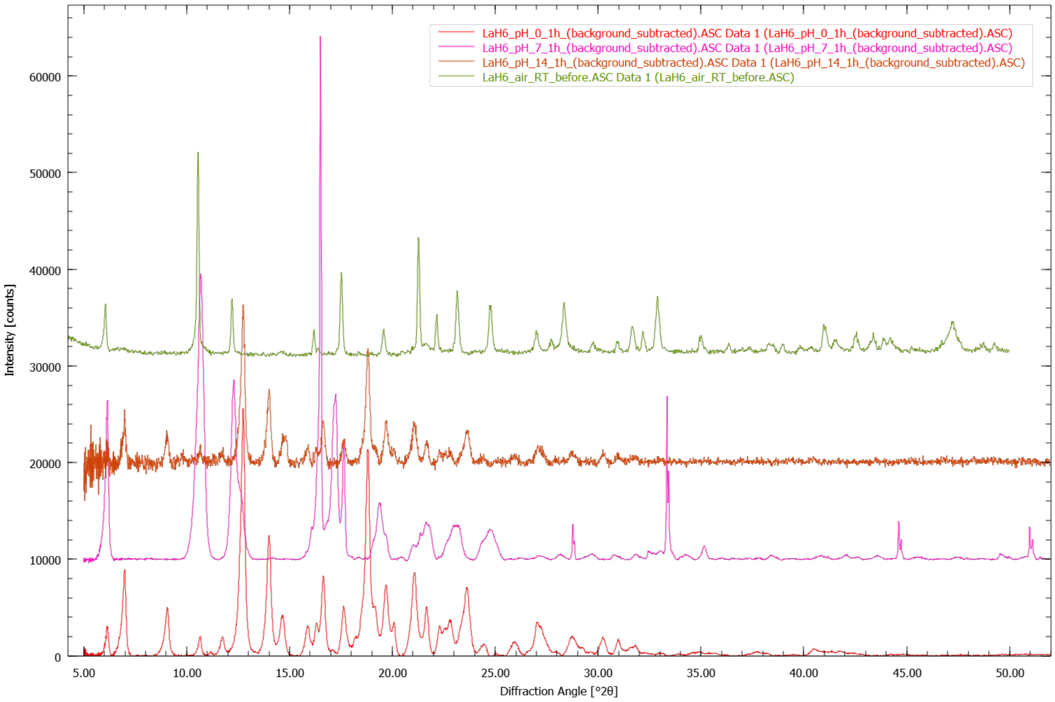

Figure S6. Chemical stability of CTH-17, pH 0 (red), pH 7 (violet), pH 14 (orange) and as-synthesized (green).

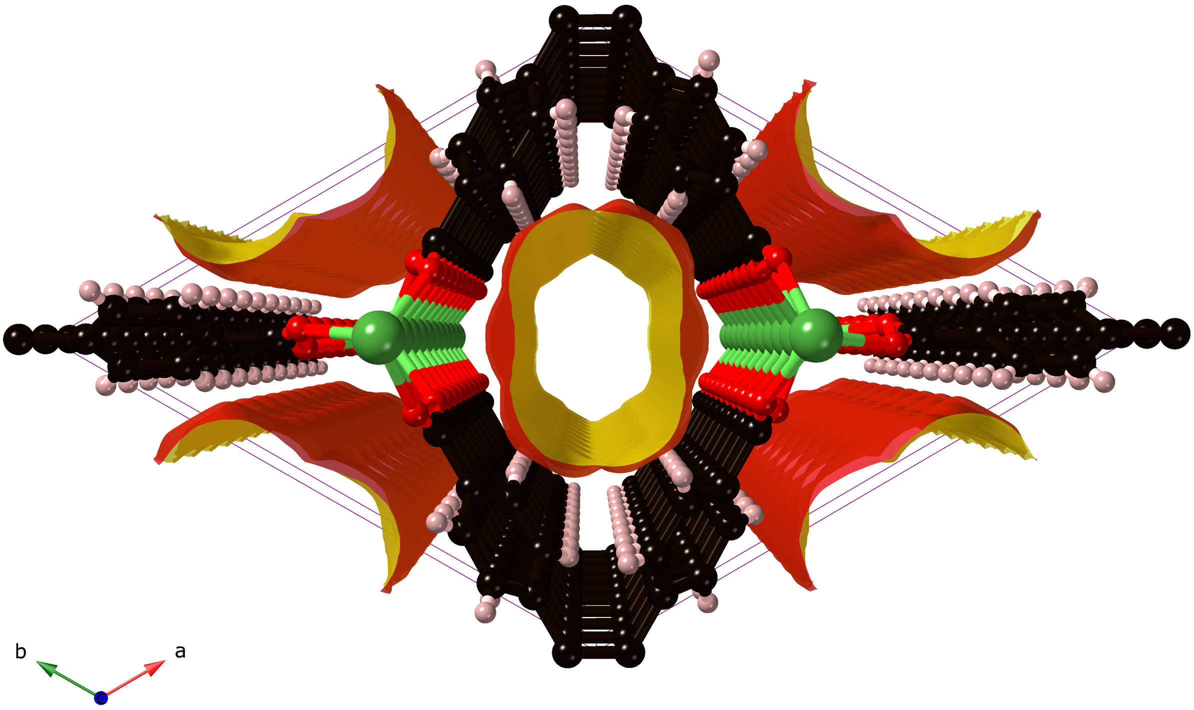

Figure S7. Visualization of the channels in **CTH-17**. There are three of these for each unit cell.

Network topology

[La_2(cpb)]

The STR (straight rod) approach,[3-4] gives the **yav**-net, a 8-connected net combining hexagons and trigonal bipyramids, just as the acetate bridged dot-MOFs CTH-7 and 8, [M_4(cpb)(acetato)$_2$(dmf)$_4$] with Fe(II) or Co(II). The more elaborate points-of-extension method gives a new 5,6-c net where trigonal prisms share triagonal faces. The standard method, ignoring the rod-structure gives the 6,12-c **alb**-net. And the points of extension and metal centers (PE&M)[1], gives a three nodal 6,6,12-c net.

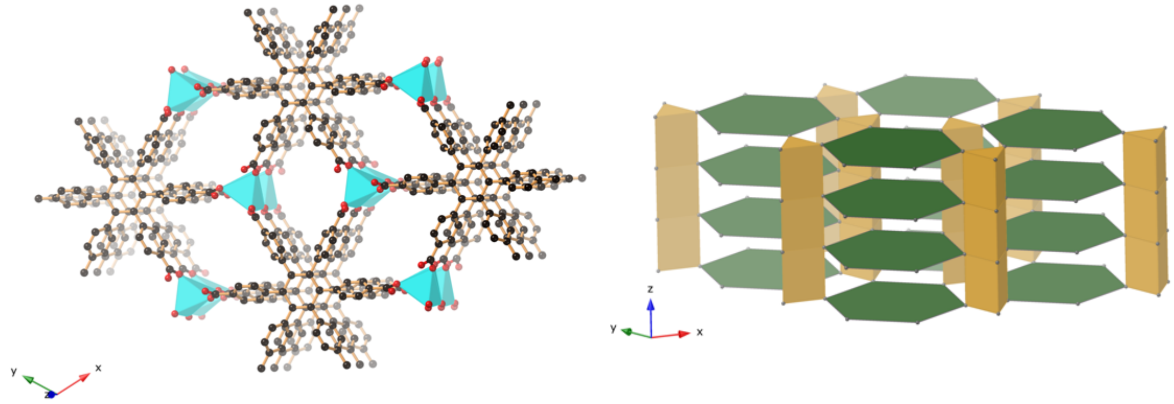

Figure S8. CTH-17 and a representation of the face-sharing trigonal prisms and hexagons of the **och** net.

SYSTRE input and output

```
Data file
"/Users/lars/Documents/Manuskript/FrancoiseLaH6/LaH6CTH15net.cgd".
LaH6CTH17net.cgd
CRYSTAL
  NAME Axel#1
  GROUP P622
  CELL 17.2930 17.2930 5.53 90.0000 90.0000 120.0000
  NODE 1 5  0.7500 0.2500 0.5000
  NODE 2 6  0.00000 0.00000 0.50000
EDGE 1 0.7500 0.2500 1.5000
EDGE 1 0.7500 0.2500 -0.5000
EDGE 1 0.5000 0.2500 0.5000
EDGE 1 0.7500 0.5000 0.5000
EDGE 1 1.0000 0.0000 0.5000
# EDGE_CENTER  0.00000 0.00000 0.18765
END

Structure #1 - "Axel".
   Input structure described as 3-periodic.
   Given space group is P622.
   7 nodes and 18 edges in repeat unit as given.
   Given repeat unit is accurate.
   Point group has 24 elements.
   2 kinds of node.
   Coordination sequences:
      Node 1:     5 15 40 75 111 166 231 291 376 471
      Node 2:     6 24 44 74 128 170 224 314 380 458
   TD10 = 1788
   Wells point symbols:
      Node 1:   3.4^4.6^5
```

```
      Node 2:    6^15
   Ideal space group is P6/mmm.
   Ideal group or setting differs from given (P6/mmm vs P622).
   Structure is new for this run.
   Relaxed cell parameters:
       a = 2.73206, b = 2.73206, c = 0.99999
       alpha = 90.0000, beta = 90.0000, gamma = 120.0000
   Cell volume: 6.46410
   Relaxed positions:
      Node 1:     0.21132 0.42265 0.00000
      Node 2:     0.00000 0.00000 0.00000
   Edges:
      0.21132 0.42265 0.00000   <->   0.21132 0.78868 0.00000
      0.21132 0.42265 0.00000   <->   0.21132 0.42265 1.00000
      0.00000 0.00000 0.00000   <->   0.21132 -0.21132 0.00000
   Edge centers:
      0.21132 0.60566 0.00000
      0.21132 0.42265 0.50000
      0.10566 -0.10566 0.00000

   Edge statistics: minimum = 0.99999, maximum = 1.00000, average = 1.00000
   Angle statistics: minimum = 60.00000, maximum = 180.00000, average =
108.00000
   Shortest non-bonded distance = 0.99999

   Degrees of freedom: 3

Finished structure #1 - "Axel".
```

[La(HCO_2)$_3$]

The STR approach, [3-4] gives the **fnu**-net, a 5-connected net with straight rods based on the axial parts of trigonal bipyramids and the trigonal parts rotated 60° between layers. But this is really a 3D SBU.

The more elaborate points-of-extension method gives a new uninodal 10-c net know to topos as 10T22 made from corner sharing elongated square bipyramids.

The standard method, ignoring the rod-structure gives a new 3,9-c net know to Topos as ith-3,9-R3m and identified in the isostructural Ce(III) compound. The points of extension and metal centers (PE&M) approach is in this case identical to the standard method.

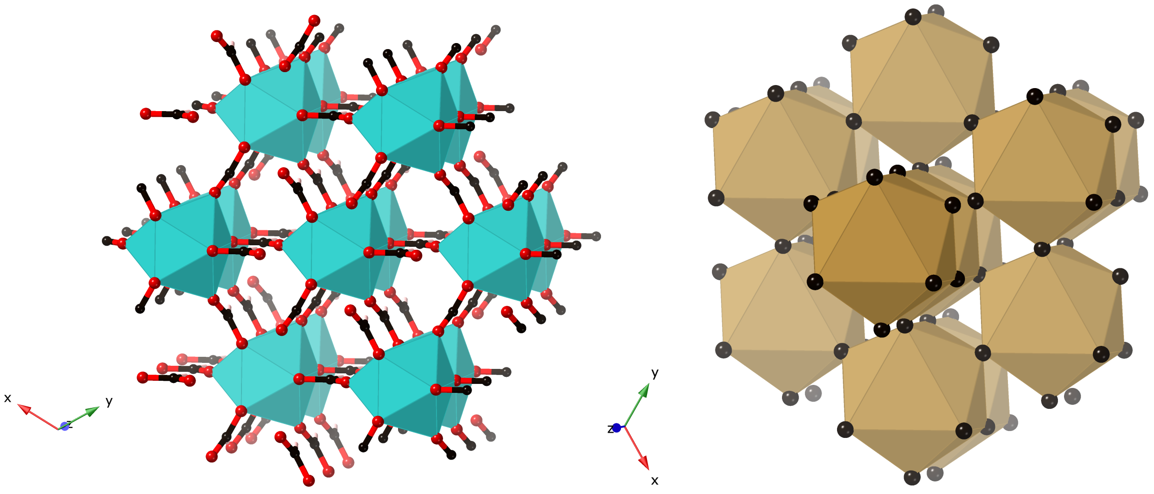

Figure S9. [La(HCO_2)$_3$].

```
CRYSTAL
  NAME Laformate
  GROUP R3m
  CELL 10.7232 10.7232 4.14 90.0000 90.0000 120.0000
  NODE 1 10  0.51428 0.48572 0.610467
END

Input structure described as 3-periodic.
   Given space group is R3m.
   3 nodes and 15 edges in repeat unit as given.
   Given repeat unit is accurate.
   Point group has 12 elements.
   1 kind of node.
   Coordination sequences:
      Node 1:    10 34 82 144 222 330 430 592 706 930
   TD10 = 3481
   Wells point symbols:
      Node 1:   3^18.4^18.5^9
   Ideal space group is R-3m.
   Ideal group or setting differs from given (R-3m vs R3m).
     (using hexagonal setting)
   Structure is new for this run.
   Relaxed cell parameters:
       a = 3.03818, b = 3.03818, c = 0.96066
       alpha = 90.0000, beta = 90.0000, gamma = 120.0000
   Cell volume: 7.67942
   Relaxed positions:
      Node 1:    0.00000 0.50000 0.00000
   Edges:
      0.00000 0.50000 0.00000  <->  0.00000 0.50000 1.00000
      0.00000 0.50000 0.00000  <->  0.16667 0.33333 -0.66667
      0.00000 0.50000 0.00000  <->  0.16667 0.33333 0.33333
   Edge centers:
      0.00000 0.50000 0.50000
      0.08333 0.41667 -0.33333
      0.08333 0.41667 0.16667
   Edge statistics: minimum = 0.93368, maximum = 1.08599, average = 1.00000
   Angle statistics: minimum = 53.86207, maximum = 180.00000, average =
100.00000
   Shortest non-bonded distance = 1.51909
   Degrees of freedom: 2
Finished structure #1 - "Laformate".
```

References

1. P.Kistaiah, K.S.Murthy, L.Iyengar, K.V.K.Rao, X-ray studies on the high pressure behaviour of some rare-earth formates, *J.Mater.Sci.* **1981**, 16, 2321

2. Bolotovsky, R. L.; Bulkin, A. P.; Krutov, G. A.; Kudryashev, V. A.; Trunov, V. A.; Ul'yanov, V. A.; Antson, O.; Hiismäki, P.; Pöyry, H.; Tiitta, A.; Loshmanov, A. A.; Furmanova, N. G., Neutron diffraction study of the crystal structure of rare-earth and yttrium anhydrous deuterated formates. *Solid State Commun.* **1990,** *76* (8), 1045-1049.

3. Xie, L. S.; Alexandrov, E. V.; Skorupskii, G.; Proserpio, D. M.; Dincă, M., Diverse π–π stacking motifs modulate electrical conductivity in tetrathiafulvalene-based metal–organic frameworks. *Chem. Sci.* **2019,** *10* (37), 8558-8565.

4. Tshuma, P.; Makhubela, B. C. E.; Öhrström, L.; Bourne, S. A.; Chatterjee, N.; Beas, I. N.; Darkwa, J.; Mehlana, G., Cyclometalation of lanthanum(iii) based MOF for catalytic hydrogenation of carbon dioxide to formate. *RSC Advances* **2020,** *10* (6), 3593-3605.
